# Scoping review of clinical decision aids in the assessment and management of febrile infants under 90 days of age

**DOI:** 10.1186/s12887-025-05619-3

**Published:** 2025-04-04

**Authors:** Etimbuk Umana, Hannah Norman-Bruce, Thomas Waterfield

**Affiliations:** 1 Wellcome Wolfson Institute of Experimental Medicine, Queen’s University Belfast, 97 Lisburn Rd, Belfast, BT9 7BL UK; 2 Emergency Department, Royal Belfast Hospital for Sick Children, Belfast, UK

**Keywords:** Bacterial infection, Febrile infant, Clinical decision aid

## Abstract

**Background:**

Clinical decision aids (CDA) play an important role in the management of young febrile infants (under 90 days of age) who are at risk of serious or invasive bacterial infections (SBI/IBI). Since 2010, a number of tailored CDAs have been developed that allow for lower-risk infants to be managed safely while undergoing fewer investigations and not receiving parenteral antibiotics. We aimed to map the CDAs developed since 2010, their derivation methodology, and their variable components.

**Methods:**

A scoping review based on the Joana Briggs Institute framework was conducted for studies published between 2010 and 2025. A database search was conducted using Medline, Embase, Scopus, Web of Science, Google Scholar, and the Cochrane library. Studies evaluating the derivation, validation, and application of CDAs for the assessment of febrile infants were eligible for inclusion. Two reviewers independently screened, analysed, and extracted data from the literature.

**Results:**

A total of 32 studies met the inclusion criteria. The majority of studies were conducted in North America and Canada (56%), followed by Europe (28%), and Asia (16%). Of the 32 studies, 14 were retrospective, 9 prospective and 9 secondary analysis of an available dataset. There were 32 CDAs that were either derived or validated across 32 studies. The derivation methodology was classified into four themes: (i) expert consensus and evidence synthesis; (ii) regression analysis; (iii) recursive partitioning; and (iv) machine learning. CDAs typically either identified a low-risk cohort through sequential assessment (*n* = 12) or predicted the risk of IBI/SBI using prediction models (*n* = 20). CDA sensitivity and specificity ranged from 46 – 100% and 9 – 95% respectively for SBI/IBI. The majority (*n* = 18) of the more complex CDA prediction models have been published in the last five years. The most common variables included within the CDAs were age, urinalysis, height of fever, C-reactive protein, and absolute neutrophil count.

**Conclusion:**

This scoping review highlights a wide range of CDAs with a trend towards prediction modelling rather than sequential assessment in the last five years. There is still variability in CDA properties, applicability, and diagnostic performance, necessitating further validation of common CDA and prediction models.

**Supplementary Information:**

The online version contains supplementary material available at 10.1186/s12887-025-05619-3.

## Introduction

Clinical decision aids (CDAs) have been developed to assist clinicians in the management of febrile infants under 90 days of age [1–3]. Some of these come in the form of risk scores, clinical prediction rules, clinical criteria, and clinical practice guidelines. Febrile infants are a challenging cohort to manage as signs and symptoms of invasive bacterial infection (IBI) and or urinary tract infection (UTI) (previously together termed serious bacterial infection (SBI)) might not be apparent [4–7]. Therefore, clinicians rely on guidance in terms of managing this cohort.

Over the last 30 years CDAs for this cohort have evolved, with earlier CDAs (Pre 2010) derived from expert consensus such as the Rochester, Boston, Milwaukee, and Philadelphia criteria [8–11]. These CDAs were developed to identify febrile infants at low risk of SBI who could be managed as outpatients with or without antibiotics. The sensitivity and specificity of the four CDAs ranged from 84% – 100% and 27%—69% respectively [8–11]. The Rochester, Milwaukee and Philadelphia CDAs focused mostly on infants less than 60 days of age, whilst the Boston criteria evaluated febrile infants between 28 and 90 days of age [11–14]. The studies on which these four early CDAs are based were undertaken in urban EDs in the USA, prior to child vaccinations for Haemophilus influenzae, Streptococcus pneumoniae, and Neisseria meningitidis. These early CDAs have been used for subsequent guideline development in North America and internationally. These CDAs have been extensively evaluated in reviews by Hui et al. and Huppler et al. [1, 2].

Over the last decade newer CDAs have been developed to reflect changes in epidemiology and diagnostic testing [12–17]. These new CDAs have been derived using more complex methodologies such as regression analysis and machine learning [18–21]. These newer CDAs use either clinical factors or laboratory biomarkers with some using a combination of both [12–15, 22–24]. Some of the CDAs use sequential assessment generating a low-risk criteria while other use more complex methods (machine learning) and give risk estimates [12–15, 22–24]. This could affect how these CDAs are interpreted or applied by clinicians in another jurisdiction and different settings. Since, the last published reviews [1, 2] there has been no subsequent review of newly published CDAs in the field of febrile infant management. The aim of this scoping review is to map published CDAs that have been derived and/or validated since 2010. The objectives of this review are as follows.

1. To report the current derived/validated CDAs used for the assessment of young febrile infants.
2. Report the derivation methodology used for each CDA.
3. Describe the variables used for each CDA
4. Report the variations in definitions of UTI, IBI and SBI
5. To report Incidence of IBI, UTI and SBI
6. Summarise the performance of the CDAs

## Methods

This scoping review followed the framework proposed by the Joanna Briggs Institute [25]. Additionally, the Preferred Reporting Items for Systematic Reviews and Meta-Analysis extension for scoping reviews (PRISMA-ScR) was used to guide the reporting of the scoping review [26]. The methodological frameworks for performing a scoping review involved the following stages: define the research question, identify relevant published literature, select relevant literature, extract and chart the data collected from the literature, and summarise and report the results. Ethical approval was not required.

### Eligibility criteria

Studies (both retrospective and prospective) examining the derivation, validation, and application of CDAs to the assessment of febrile infants were eligible for inclusion. Participants of included studies were febrile infants less than 90 days of age who had attended an acute care service (emergency department or assessment unit) within 48 h of the onset of fever measured by any thermometer either in acute care setting or at home.

The reference standard for the included studies was IBI or SBI according to the individual study authors. As a guide from the existing literature, IBI is defined as the isolation of a bacterial pathogen in blood or cerebrospinal fluid (CSF) culture or using a quantitative PCR assay. SBI, which is less standardised thus far in the literature, can be inclusive of IBI, UTI, pneumonia, gastroenteritis, and other bacterial infections.

Studies outside the predefined age range or where information on the diagnostic accuracy of the CDAs could not be extracted from the published manuscript were excluded. Studies where IBI or SBI was not the primary reference standard and studies focusing solely on biomarkers were also excluded. Owing to the resource and time constraints of this review, non-English studies were excluded. Case reports, systematic reviews, cost-effectiveness analyses, quality improvement projects, editorials, and letters were excluded.

### Search strategy

A search was conducted using a broad selection of MeSH terms and keywords related to the objectives and research questions:

Bacterial infection* OR invasive bacterial infection* OR serious bacterial infection* (MeSH terms) And (Fever OR febrile) And (clinical decision aid OR prediction rule OR prediction model OR learning model OR risk stratification OR clinical criteria OR clinical score OR clinical practice guideline (Key terms).

The search was limited by age, utilising database limits (infants or newborns). An example search output can be found in Supplementary File 1. A pilot search was undertaken using Medline by EU, who subsequently reviewed 10 publications to assess the search strategy was generating appropriate publications. Following this piloting process, a full search was conducted in the following databases: Ovid Medline, Embase, Scopus, Web of Science, Cochrane Library, and Google Scholar (for grey literature). The search strategy was limited to 2010 as two previous systematic reviews (Hui et al. and Huppler et all) have extensively covered older CDAs prior to 2010 [1, 2]. References of the included studies were also searched for additional publications.

### Screening and data extraction

Initial screening was performed by two authors (EU and HNB) independently based on the title and abstract to ensure that they fulfilled the eligibility criteria. This process was undertaken using Rayyan’s online management programme [27]. Studies not meeting the eligibility criteria and duplicate studies were excluded from further review. The remaining studies underwent a full-text analysis by the same two independent reviewers, and any discrepancies were resolved by a third author (TW). A data extraction template was developed and piloted by the extraction team (EU and HNB) (Supplementary File 2). Data were then extracted, stored, and charted using a Microsoft Excel spreadsheet. The extracted data included study characteristics (design, year of publication, journal, year study was conducted, sample size, and country), participant characteristics (age, sex, fever with a source (FWAS)), CDA components and derivation methodology, reference standards (IBI and SBI), and outcomes (diagnostic accuracy). FWAS (defined as temperature measured at home or at the ED ≥ 38 °C in patients with an abnormal physical examination and respiratory signs/symptoms or a diarrhoeal process) [12].

### Data synthesis

The extracted data were collected and are represented in tables, graphs, and figures. The CDAs were grouped into those using low-risk criteria and prediction models (without low-risk criteria). Where a CDA had been validated in more than one study, the data were aggregated, and the diagnostic performance was reported as a range. The CDA derivation methods were compared and analysed based on themes.

## Results

**Fig. 1:**
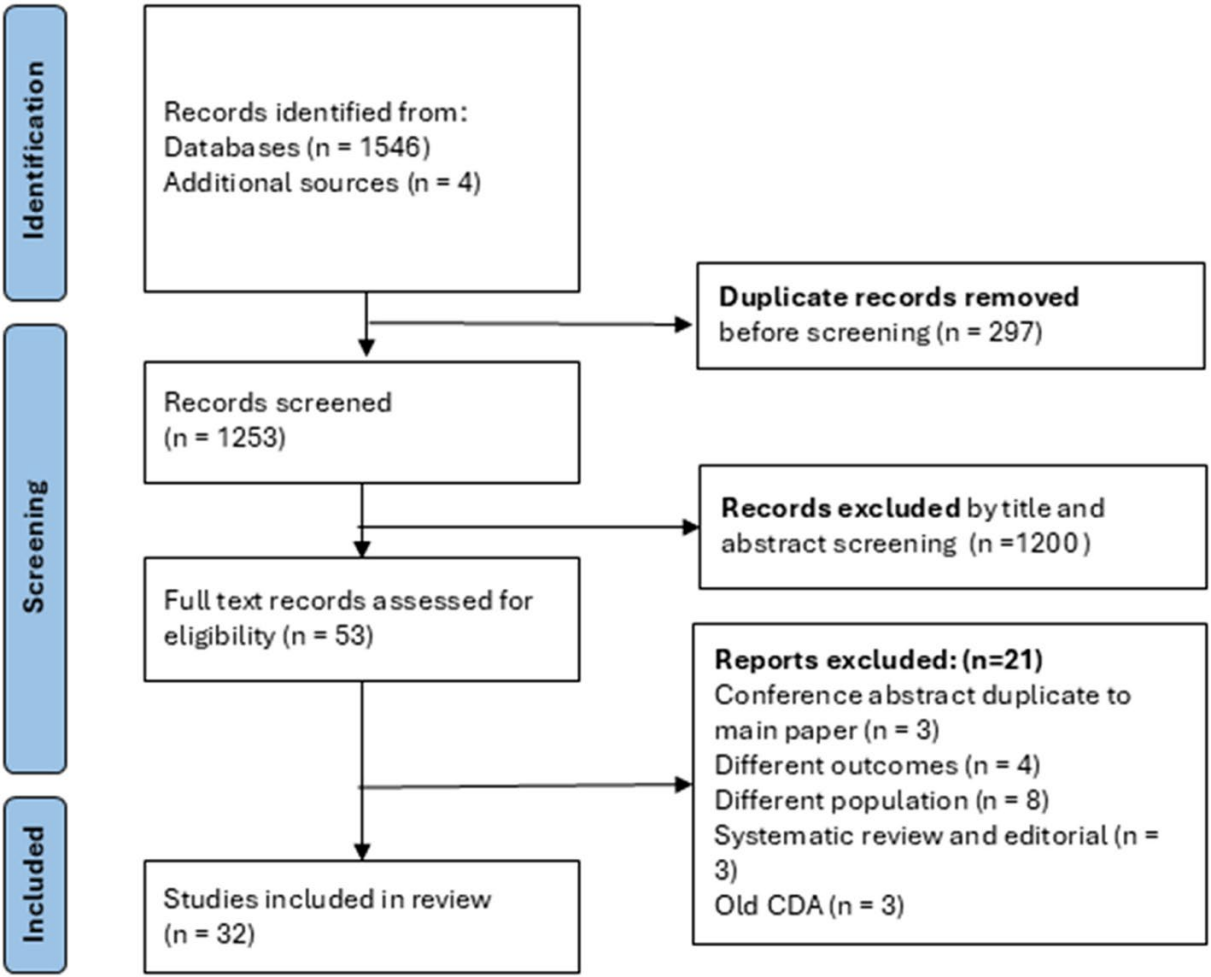
PRISMA flow diagram of scoping review [28]

**Fig. 2:**
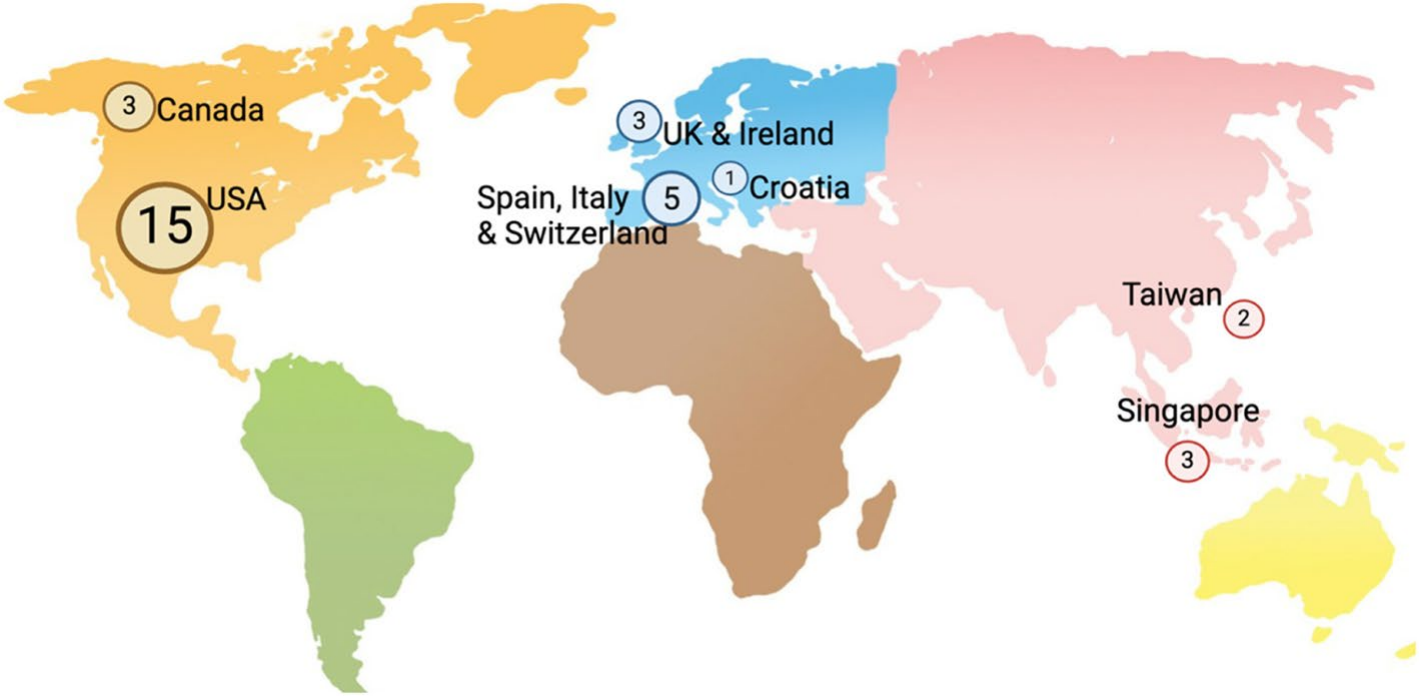
Frequency of CDA’s origin

A database search identified 1550 publications from January 2010 to February 2025. After title and abstract reviews, 53 studies underwent a full-text review. Of these, 32 met the inclusion criteria for this scoping review (Fig. 1). Majority (*n* = 18) of studies were performed in North America, followed by Europe (*n* = 9) and Asia (*n* = 5) (Fig. 2). Funding sources was reported in 14 (45%) studies. Of the 32 studies, 14 were retrospective, 9 were prospective, and 9 were secondary analyses of an available dataset. Infants with FWAS were excluded in 5 studies. The number of sites per study ranged from 1 to 37, and the sample size ranged from 181—4411 (Table 1). Most studies focused on a population aged 0 – 90 days (*n* = 14) or 0–60 days (*n* = 8) of age, whilst 9 studies excluded infants less than a week old.

**Table 1:** Study characteristics summary

Study Variables	N (% of total 31 studies)
Region of study origin	
Asia	5 (16%)
Europe	9 (28%)
North America	18 (56%)
Funding declared	
Yes	15 (47%)
No	16 (50%)
NR	1 (3%)
Study design	
Prospective	9 (28%)
Retrospective	14 (44%)
Secondary Analysis	9 (28%)
Studies where “FWAS” excluded	
Yes (FWAS excluded)	5 (16%)
No, (FWAS included)	27 (84%)
Age of infants	
0—56 days	1 (3%)
0—60 days	8 (25%)
0—90 days	14 (44%)
7—60 days	3 (9%)
8—60 days	4 (13%)
8—90 days	2 (6%)
Sample size per study	
Median (IQR)	1111 (602–1930)
Range	181 – 4411
Number of sites per study (30 recorded)	
Median (IQR)	3 (1 – 11)
Range	1 – 37
Reported outcomes of study	
IBI	17 (53%)
SBI	11 (34%)
IBI + SBI	4 (13%)
Gender (29 studies reported)	
Male < 50%	2 (7%)
Male > 50%	27 (93%)
Incidence of IBI (28 studies)	
Range	1.6%—5.9%
Meningitis	- 1%
Incidence of SBI (21 studies)	
Range	7%—38.7%
UTI (21 studies)	5.7 – 29.8%

*NR* Not recorded, *IBI* Invasive bacterial infection, *SBI* Serious bacterial infection, *UTI* Urinary tract infection, *FWAS* Fever with a source, *IQR* Interquartile range

### IBI and SBI

17 studies had IBI as their main outcome compared to 11 and 4 studies which had SBI and SBI + IBI as their main outcome respectively. The incidence of IBI was obtained from 28 studies and ranged from 1.6% to 5.9% (Table 1). The incidence of SBI ranged from 7 to 39% (21 studies). The authors’ definition of IBI was consistent across studies compared to that of SBI which showed marked variation (Table 2).

**Table 2:** Author definition of IBI and SBI

Study Definition	N (% of 28 studies)
Author IBI Definition	
Bacterial Meningitis and Bacteraemia	24 (75%)
Bacterial Meningitis and Bacteraemia + Paediatric infectious disease review	2 (6%)
NR	6 (19%)
Author SBI Definition	
Bacterial Meningitis, Bacteraemia and UTI	15 (47%)
Bacterial Meningitis, Bacteraemia, UTI and Bacterial Gastroenteritis	3 (9%)
Bacterial Meningitis, Bacteraemia, UTI, Bacterial Gastroenteritis, Pneumonia and Cellulitis	1 (3%)
Bacterial Meningitis, Bacteraemia, UTI, Bacterial Gastroenteritis, Pneumonia, Osteomyelitis, Septic Arthritis and Soft Tissue Infection	1 (3%)
UTI and Bacterial Gastroenteritis	1 (3%)
NR	11 (34%)

*NR* Not recorded, *IBI* Invasive bacterial infection, *SBI* Serious bacterial infection, *UTI* Urinary tract infection

### CDA and derivation methodology

**Fig. 3:**
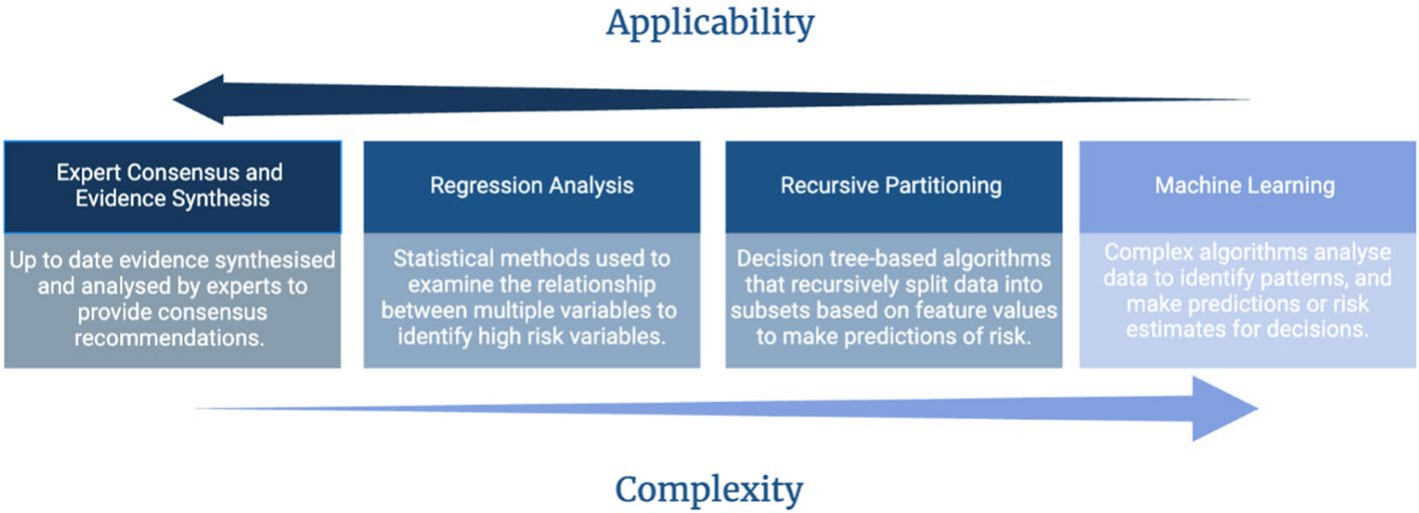
Summary of CDA derivation methodology used across all 32 studies

There were 32 documented CDAs, which were either derived or validated across 32 studies. 15 of the CDAs were derived from the USA, while the others were derived from Spain, the UK, Croatia, Taiwan, and Canada (Supplementary File 3). As described above, CDA were also classified into two categories: those using low-risk criteria [12, 13, 15, 16, 18, 29, 30] and those using prediction models [19–23, 31–36] (Tables 3 and 4). In addition to these two types of CDA, the derivation methodology was analysed and grouped into 4 main themes. Namely, (i) expert consensus + evidence synthesis, (ii) regression analysis, (iii) recursive partitioning, and (iv) machine learning (Fig. 3). All CDAs using low risk criteria (*n* = 12) were derived after 2010 with the exception of the Lab-score which was derived in 2008 for children under 24 months old but only validated in the under 90 days in the post 2010 era [29, 37]. Most (*n* = 18) CDA prediction models have been published in the last 5 years (2020 – 2025) (Supplementary File 4). Only 5 CDA of the prediction model studies reported either probability levels or risk thresholds as the outputs of their models (Table 3).

**Table 3:** Diagnostic performance of CDA Prediction models

**CDA Prediction Models**	**Study**	**Year of derivation**	**Derivation Methodology**	**Probability cutoff**	**Risk treshold**	Sensitivity SBI	Specificity SBI	PPV SBI	NPV SBI	Sensitivity IBI	Specificity IBI	PPV IBI	NPV IBI	AUC	Validation (Number of studies)
Regression Analysis [19]	VIllalobos-2017	2017	Expert consensus + binary logistic regression	80	N/A	87.7%	70.1%	-	-	-	-	-	-		0
Regression Analysis + AIC [31]	Vujevic-2017	2017	Logistic Regression and Akaike Information Criterion (AIC)	40	N/A	74.3%	88.3%	-	-	-	-	-	-		0
Step wise regression [20]	Ramgopal-2020 (Derivation and Validation)	2020	Regression	N/A	N/A	98.6—100%	49.2—50%	16.7—17%	99.7—100%	-	-	-	-	0.95	1
Random Forest Modelling [20]	Ramgopal-2020 (Derivation and Validation)	2020	Machine learning	N/A	N/A	99—100%	75—82%	29—36%	100%	-	-	-	-	0.96	1
Support Vector Machine Model [20]	Ramgopal-2020 (Derivation and Validation)	2020	Machine learning	N/A	N/A	97%	48—52%	16—17%	99%	-	-	-	-	0.93	1
Single-Hidden Layer Neural Network [20]	Ramgopal-2020 (Derivation and Validation)	2020	Machine learning	N/A	N/A	96—99%	69—72%	24-—26%	99%	-	-	-	-	0.95	1
Logistic Regression [32]	Chiu-2021	2021	Regression	N/A	N/A	-	-	-	-	90.0%	59.0%	-	-	0.85	0
Support Vector Machine Model [32]	Chui-2021	2021	Machine learning	N/A	N/A	-	-	-	-	91.0%	60.0%	-	-	0.84	0
Extreme Gradient Boosting [32]	Chui-2023	2021	Machine learning	N/A	N/A	-	-	-	-	90.0%	57.0%	-	-	0.84	0
Not Documented [33]	Poirier-2021	2021	Not Documented	N/A	N/A	90%	32.9%	-	98.1%	100%	30.0%	-	100%		0
Regression Analysis [22, 23, 38]	Yaeger 2021 – 2022 (Derivation and Validation)	2021 & 2022	Regression	N/A	0.01/0.03/0.05	92.9—94.6%	50—74.5%	16.1- 23.9%	99%	86.4—94.9%	47.3—51.6%	3.3- 20.5%	99%	0.92–0.95	1
Super Learner Modelling [22, 23, 38]	Yaeger 2021 – 2022 (Derivation and Validation)	2021 & 2022	Machine learning	N/A	0.01/0.03/0.06	96%	65—74%	17—23%	99.5%	91—100%	3.4—51%	2—3%	99.7—100%	0.9–0.96	1
FIRST [35]	Chong-2023	2023	AutoScore Machine Learning Model	N/A	5%/10%/15% >	93.2% (Predicted Risk-15%)	29.9%	27.5%	94%	-	-	-	-	0.74	0
FIRST + [35]	Chong-2023	2023	AutoScore Machine Learning Model	N/A	5%/10%/15% >	81.8% (Predicted Risk-15%)	65.6%	40.4%	92.7%	-	-	-	-	0.88	0
HRV and HRnV [34]	Chong-2023	2023	Regression	N/A	N/A	-	-	-	-	-	-	-	-	0.81	0
Deep Learning Model [21]	Yang-2023 (Derivation and Validation)	2023	Machine learning	N/A	N/A	-	-	-	-	99—100%	48—54%	5—6%	100%	0.87	1
LASSO [36]	Ballard 2024	2024	Machine Learning	N/A		-	-	-	-	-	-	-	-	0.83	0
Logistic [36]	Ballard 2024	2024	Machine Learning	N/A		-	-	-	-	-	-	-	-	0.84	0
Random Forest [36]	Ballard 2024	2024	Machine Learning	N/A		-	-	-	-	-	-	-	-	0.81	0
XGBoost [36]	Ballard 2024	2024	Machine Learning	N/A	< 1%/ < 2%/ < 3%/ < 5%/ < 10%	-	-	-	-	-	-	-	-	0.84	0

*FIRST* Febrile infants risk score at triage, *LASSO* Least absolute shrinkage and selection operator, *N/A* Not applicable, *CDA *Clinical decision aid, *IBI *Invasive bacterial infection, *SBI* Serious bacterial infection, *PPV* Positive predictive value, *NPV* Negative predictive value, *AUC* Area under the curve

**Table 4:** Diagnostic performance of CDAs using low-risk criteria

**CDA (with low risk criteria)**	**Low risk criteria**	**Year of derivation**	**Derivation Methodology**	Sensitivity SBI	Specificity SBI	PPV SBI	NPV SBI	Sensitivity IBI	Specificity IBI	PPV IBI	NPV IBI	Validation (Number of studies)
Lab Score [12, 29, 39]	Low risk: Lab score ≤ 31 point is attributed to positive urine dipstick; 2 points for PCT ≥ 0.5 ng/mL or CRP ≥ 40 mg/L; and 4 points to procalcitonin ≥ 2 ng/mL or CRP ≥ 100 mg/L	2008	Regression Analysis	46%—52% -	92 – 95%	76%−80%	78%—83%	59.8—88.9%	84—89%	8- 15%	98—99.5%	3
Morgan Stanley Children Hospital Protocol [40]	Low risk: WBC count between 5000 and 15 000/μL, manual differential of immature: total neutrophil ratio of < 0.2, urinalysis with < 10 WBC/high-power field, normal glucose and liver function tests (If obtained: Normal CSF, Stool and CXR test.)	2012	Expert Consensus and Evidence Synthesis	97.40%	17.3%	26.1%	95.7%	-	-	-	-	1
NICE NG143 [15, 41]	> 1 month, well appearing, WCC 5–10^9/L	2019	Expert Consensus and Evidence Synthesis	91%	9%	14%	86%	93%	27%	5%	99%	2
NICE NG51 [15]	> 3 months	2016	Expert Consensus and Evidence Synthesis	100%	0	14%	N/A	-	-	-	-	1
Step by Step [12, 20, 21, 39, 42]	Well appearing, > 21 days + absence of Leukoctouria + PCT > 0.5 ng/mL + (ANC > 10 ^9/L OR CRP > 20 mg/L)	2014	Expert Consensus and Evidence Synthesis	94–98%	17–68%	23–37%	94–99%	92—100%	12.4—46.9%	4- 7.4%	99.3—100%	4
Roseville protocol [43, 44]	7—28 days: Temp < 38.5C, Normal WBC, absolute band count and urinalysis. 22—60 days: Normal WBC, absolute band count and urinalysis	2016	Expert Consensus and Evidence Synthesis	-	-	-	-	96.7% (7–28 days), 91.4% (29–60 days)	31.7% (7–28 days), 58.5% (29–60 days)	6.6% (7–28 days), 6.3% (29–60 days)	99.5% (7–28 days), 99.6% (29–60 days)	2
Aronson IBI Score [18, 20, 21, 32, 41, 45]	Low risk: IBI score of 2 Newborn < 21 d old: 1 point, Body temperature 38.0–38.4 °C: 2 points, Body temperature > 38.4 °C: 4 points, Abnormal urinalysis result: 3 points, Neutrophils > 5.185 *10^9/L: 2 points	2019	Regression Analysis	99–100%	31–32%	13%	82%	85—100%	26.6—43%	2.7—4%	99.7—100%	5
PECARN [13, 20, 39, 46, 47]	Negative urinalysis, ANC < / = 4000/uL and PCT < 0.5 ng/ml	2019	Recursive Partitioning	88.4—98.8%	36.6—64%	19.5—41.1%	86.3—99.8%	89–100%	29–63%	4–5%	99–100%	5
BSAC[15, 41]	> 1 month and well appearing, negative urinalysis, CRP < 20 mg/L	2020	Expert Consensus and Evidence Synthesis	82%	14%	13%	82%	99	20	5	100	2
AAP [16, 17, 41, 44, 48, 49]	> 21 days, appearing well, ANC < 5.2^9/L, CRP < 20 mg/L, Fever < 38.5 °C	2021	Expert Consensus and Evidence Synthesis	84%	51%	-	94%	93–100%	17—50.7%	2.7—4.3%	99.3—100%	6
AAP V2 [48]	ANC ≤ 4500/mm3, CRP ≤ 22.2 mg/L, TMax ≤ 39.0 °C	2025	Regression Analysis	-	-	-	-	100%	83.8%		100%	0
CA FIRST [50]	Aged 29—60, Well appearing, normal urinalysis + bronchiolitis	2021	Expert Consensus and Evidence Synthesis	95% (7–21 days), 100% (22–28 days), 90% (29–60 days)	27% (7–21 days), 32% (22–28 days), 56% (29–60 days)	10% (7–21 days), 7% (22–28 days), 6% (29–60 days)	99% (7–21 days), 100% (22–28 days), 99% (29–60 days)	-	-	-	-	0

*CA FIRST* California Febrile Infant Risk Stratification Tool, *Lab Score *Laboratory score, *NICE* National Institute for Health and Care Excellence, *BSAC *British Society for Antimicrobial Chemotherapy, *PECARN* Pediatric Emergency Care Applied Research Network, *AAP* American Academy of Pediatrics, *Tmax *Maximum temperature, *WBC/WCC* White blood cell count, *ANC* Absolute neutrophil count, *CRP* C-reactive protein, *PCT* Procalcitonin, *CDA *Clinical decision aid, *IBI *Invasive bacterial infection, *SBI* Serious bacterial infection, *PPV* Positive predictive value, *NPV* Negative predictive value, *AUC* Area under the curve

#### CDA performance

The CDAs using low-risk criteria and sequential assessment that have undergone more than 2 validations are the Pediatric Emergency Care Applied Research Network (PECARN), Step-by-Step, American Academy of Pediatrics (AAP), Aronson score and Lab score. The sensitivity and specificity ranges for low-risk CDA for IBI and SBI are shown in Table 4.

For CDAs based on prediction modelling, Yang; deep learning model, Yager; regression analysis and super learner model as well as Ramgopal; stepwise regression, random forest modelling, support vector machine model, and single-hidden layer neural network Models have been validated. Their performances are listed in Table 3.

### Variables in CDA

**Fig. 4:**
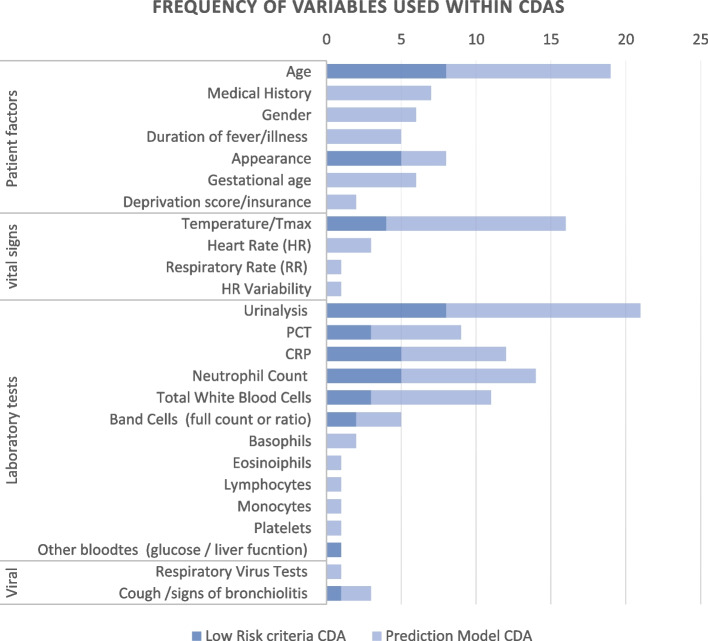
Frequency of variables used across 32 CDAs. *CRP* C-reactive protein, *PCT* Procalcitonin, *CDA* Clinical decision aid

There were 25 key variables found within the CDAs reported in this review. These variables were grouped into patient factors, vital signs, urine tests, blood tests, and viral markers (Fig. 4). The most common variables in more than 10 CDAs were age (*n* = 19), urinalysis (*n* = 21), temperature level or (*n* = 11), C-reactive protein (CRP) (*n* = 16), absolute neutrophil count (ANC) (*n* = 14) and CRP (*n* = 12). Only 9 CDA had procalcitonin (PCT) as a part of their CDA. The Yaeger regression and super learner models had the greatest number of variables in the CDA (*n* = 10). (Supplementary File 5).

## Discussion

A total of 32 Clinical Decision Aids (CDAs) have been either derived or validated since 2010, resulting from 32 studies that were conducted across three different continents. The majority of these studies (57%) were conducted in North America and Canada. These CDAs can be broadly categorized into two groups based on their methodology: those that use sequential assessment to identify a low-risk cohort, and those that use prediction modelling to estimate the risk of SBI or IBI. Despite the differences in their methodologies, several variables were consistently incorporated into the CDAs, including age, urinalysis, temperature, CRP, and ANC.

Traditionally, and even up to this current era, a treat-all approach has been employed in the management of febrile infants by certain guidelines, such as the National Institute for Health and Care Excellence (NICE) in the UK [51]. Evidence from the UK reports that up to 86—90% of febrile infants are admitted, and 68—80% receive parenteral antibiotics [15, 41]. A treat-all approach has implications, such as less time for maternal foetal bonding, increased stress with hospitalisation, antibiotic impact on the microbiome, and iatrogenic complications from procedures [24, 34]. Also, the care cost of febrile infants under 90 days of age is significant and higher than that of other age groups and using a treat all approach [24, 41, 52]. Hence, the development of CDAs has advanced over the last decade to provide tailored care for these infants.

These CDAs have been derived and validated in various high-to middle-income settings, with most having different rates of IBI or SBI, clinical practice, and physician risk thresholds [12, 13, 15, 17, 19–21, 23, 31, 32, 35, 39, 43]. There was notable heterogeneity in how studies were conducted. There was a variation in age cut-off used by studies in this scoping review. It is established that the risk of IBI decreases with age, however the risk of IBI in those 60–90 days of age is not negligible and infants are still considered as high-risk aged up to 90 days [17, 53, 54]. Five studies (16%) excluded infants presenting with fever and a clear source which was mostly classified as an infant with respiratory symptoms. Masarweh et al. found in their study of over 3000 febrile infants that the rate of SBI was 2.8% in the group with respiratory symptoms compared to 7% in the group without respiratory symptoms demonstrating that the risk of SBI in infants with source (respiratory symptoms) should also not be ignored. These examples of interstudy variability mirrors the heterogeneity identified in reviews of studies prior to 2010 when looking at older CDAs [1, 2].

In this review 25 variables were identified from the CDAs used to assess febrile infants. The most frequently occurring variables were age, appearance, temperature, urinalysis, CRP, and ANC. This is in agreement with studies examining the predictive variables for both SBI and IBI [15, 55, 56]. Some CDAs focused on patient factors and vital signs (clinical features) to facilitate risk stratification prior to any investigation [23, 34, 35]. This approach would enable clinicians to perform risk stratification of febrile infants in settings where laboratory tests are not available. Additionally, some CDAs used a combination of clinical criteria and urinalysis results [22, 23, 38, 50].

The inclusion of laboratory markers increased the sensitivity of some CDAs but at the expense of specificity [31, 35]. PCT was not frequently used in CDAs, with only 9 CDAs using it compared to 12 and 14 using CRP and ANC, respectively. This is likely an overestimate of the number of CDAs using PCT given that Ramgopal et al., had derived 4 prediction model CDAs in a single study using the same variables, including PCT [20]. PCT is not readily available universally compared to CRP and ANC which can make application or externally validation of CDAs with PCT challenging [57, 58]. Uniquely, the AAP guidelines recommended that, where PCT is not available, CRP can be used [24]. However, a recent systematic review found that at the internationally recommended cut-off CRP of 20mg/l, was inferior to PCT 0.5ng/ml for identification of IBI, and suggests that a lower cut-off of CRP should be explored for the sequential assessment of febrile infants [59].

The definition of IBI was consistently defined as the growth of a single pathogen in blood or CSF. However, in studies examining SBI, the definition included IBI but also UTI, pneumonia and other infections. This heterogeneity in definitions could impact the performance of CDAs when applied to diverse populations. Therefore, the AAP has recommended focusing on IBI as an outcome due to the heterogeneity associated with SBI [24]. Complications from the delayed treatment of IBI can be catastrophic for febrile infants. Current evidence suggests that infants with UTI and are low risk can be safely managed as outpatients on oral antibiotics [24, 60–62]. This is supported by the AAP and CA FIRST protocols [24, 50]. The incidence of IBI varied from 1.6% to 5.9% across 28 studies, while that of SBI ranged from 7% to 38.7 (21 studies). The upper limit of these values exceeded the range previously reported in literature [1, 12, 13, 15]. Such variation could be attributed to factors such as epidemiology, regional practices, and patient selection in the studies included in the analysis and highlight the importance of applying and validating the various CDAs in different contexts.

This review highlights the evolution of the modern CDAs. The methodology for the derivation of the 32 CDAs ranged from less complex methods, such as expert consensus and evidence synthesis, to more complex methods, such as machine learning (Fig. 3). CDAs that use low-risk criteria often use expert consensus, evidence synthesis, regression analysis, and recursive partitioning. These typically generate optimum cut-offs for individual variables or scores that could be used to identify a low-risk cohort of febrile infants. These CDAs are more clinically applicable and can be used at the bedside by clinicians for the sequential assessment of febrile infants. Conversely, prediction model CDAs often use more complex computational analysis, such as a random forest model, support vector machine models, and single-hidden layer neural networks [20, 21, 23, 32, 35]. The outputs of these machine learning models are in the form of probabilities or risk thresholds, which presents challenges in terms of clinical applicability because the acceptance of risk thresholds varies across different contexts, let alone between different clinicians. As highlighted in Fig. 3, with increasing complexity CDAs become less applicable. Moreover, most of these models require implementation through electronic health record systems, which are not universally available. It is worth noting that most studies exploring prediction models have been conducted in the past five years, with few undergoing external validation due to their complexity. Some prediction models, such as the Autoscore proposed by Chong et al., have attempted to overcome this by including regression analysis [35]. They derived the FIRST and FIRST + scores using this method and attempted to provide a score cutoff with associated clinical risk thresholds for SBI to facilitate clinical implementation.

The diagnostic performance of the 12 CDAs using low-risk criteria varied widely for both the detection of SBI and IBI. For SBI, NICE NG51 had the highest sensitivity (100%), but the specificity was 0%. As discussed previously, this CDA supports a treat-all approach which has significant health and cost implications [41]. In contrast, PECARN CDA had a better specificity ranging from 36 to 64% compared to the other CDA using low-risk criteria for SBI. For IBI, The AAP CDA had a sensitivity of 100%, but with a specificity ranging from 17% to 50.7% However, Burstein et al. have improved this specificity to 83% using statistically derived variables for temperature, CRP and ANC [48]. Regardless, the AAP was inferior to the lab score which had the best specificity (88–89%) for IBI of the CDAs using low-risk criteria. The AAP focused on both clinical factors (age, appearance, and temperature) and laboratory markers (ANC, CRP, and PCT) compared to the Lab score, which uses only laboratory markers (urinalysis, CRP, and PCT) in the risk assessment of febrile infants. In this review, 5 of the 6 studies used CRP and ANC as laboratory markers, and 1 study used only ANC. This suggests that the AAP CDA performs effectively in diagnosing IBI even without the use of PCT. The most validated CDAs, supported by extensive evidence, are PECARN, AAP, Step-by-Step, and the Aronson score. When the studies were grouped together, there was a significant variation in the sensitivity and specificity for PECARN, Step-by-Step, Aronson score, and AAP. This underscores the challenges mentioned earlier in obtaining consistent results when validating CDAs. Therefore, unless a CDA is to be validated in the exact same population, it is recommended that the terminology be changed to "application." By using this term, the CDA can be adapted to a different jurisdiction, taking into account the local population and variations in epidemiology.

For the prediction model CDAs, sensitivities were also varied and were as low as 70%, but specificity tended to be higher than CDA using low-risk criteria for SBI. The Random Forest Modelling from Ramgopal et al. had the best sensitivity (99%—100%) and Regression Analysis + AIC from Vujevic et al. had the best specificity (88%) for SBI compared to other prediction models for SBI [20, 31]. For IBI, Yang et al. (deep learning model) and Poirier et al. achieved the best sensitivity (100%) [21, 33]. The prediction model CDA with the best specificity (60%) for IBI was developed by Chiu et al. (support vector machine learning model) [32]. Notably prediction model CDAs for IBI performed better in terms of the sensitivity. However, as the incidence of IBI is low, this can create challenges for the development of prediction model CDAs, necessitating extra statistical analysis to adjust for the low incidence [32]. Also, some prediction model CDAs focused more on the identification of high-risk infants that required immediate treatment, as opposed to low-risk infants that could be discharged home [21, 23, 34, 35]. Of the 20 CDA prediction models, only Yager et al.’s regression analysis and super learner model had undergone validation in a separate cohort [38]. Ramgopal et and Yang et al. had conducted internal validation of their prediction models within the same study [20, 21]. Although prediction models derived from machine learning represents a robust methodology for producing CDA, their external validation is challenging. Despite the rapid advancement of machine learning for the derivation of prediction models in this arena over the last 5 years, very few have undergone external validation representing an important research priority.

This review focused on the management of febrile infants presenting to hospital with a fever to whom CDA’s should be applied. This was achieved by excluding studies focusing only on neonatal sepsis (0 – 7 days) and older children up to 16 years as they represent a more varied population. As this was a scoping review, the studies were not assessed for the risk of bias. Some of the classifications and categorisations of CDAs used in this review have not been reported in the literature but were developed during the synthesis of the included articles by the authors to enable better reporting. As only studies published in English were included, this might limit the generalisability to other parts of the world with publication in other languages.

## Conclusion

Since 2010, numerous CDAs have been developed, with some validated and applied in various jurisdictions. Low-risk CDAs are more clinically useful and simpler in derivation than those from machine learning models. The incidence and definition of SBI vary, affecting their performance depending on the context. As IBI definitions are more consistent, future studies should focus on IBI and avoid using SBI. There is still variability in CDA properties, applicability, and diagnostic performance, necessitating further validation of common CDA and prediction models.

## Supplementary Information

Supplementary Material 1.

Supplementary Material 2.

Supplementary Material 3.

Supplementary Material 4.

Supplementary Material 5.

## Data Availability

All data generated or analysed during this study are included in this published article.

## Abbreviations

SBI: Serious bacterial infection
IBI: Invasive bacterial infection
UTI: Urinary tract infection
CDA: Clinical decision aids, prediction model
