# Supplementary material for: Scoping review of clinical decision aids in the assessment and management of febrile infants under 90 days of age
Source: BMC Pediatr. 2025 Apr 4;25:274. doi: 10.1186/s12887-025-05619-3 (PMC11969967; doi:10.1186/s12887-025-05619-3)
Supplement: Supplementary file 1 — Supplementary Material 1. [file 12887_2025_5619_MOESM1_ESM.docx]

Supplementary File 1. Database search example

**Embase <1974 to 2023 August 22>**

1 exp bacterial infection/ 952899

2 invasive bacterial infection*.mp. 760

3 serious bacterial infection*.mp. 1897

4 exp fever/ 333556

5 febrile.mp. 97218

6 clinical decision aid.mp. 127

7 prediction rule.mp. 2500

8 prediction model.mp. 33777

9 learning model.mp. 15016

10 risk stratification.mp. 78209

11 clinical criteria.mp. 18597

12 clinical score.mp. 7222

13 clinical practice guideline*.mp. 28638

14 1 or 2 or 3 953428

15 4 or 5 402065

16 6 or 7 or 8 or 9 or 10 or 11 or 12 or 13 180492

17 14 and 15 and 16 617

18 limit 17 to english language 590

**19 limit 18 to yr="2010 -Current" 487**

**Updated Search**

**20 limit 18 to yr= “22 August 2023 – 02 Feb 2025” 161**

**Total: 648**
