# Supplementary material for: Scoping review of clinical decision aids in the assessment and management of febrile infants under 90 days of age
Source: BMC Pediatr. 2025 Apr 4;25:274. doi: 10.1186/s12887-025-05619-3 (PMC11969967; doi:10.1186/s12887-025-05619-3)
Supplement: Supplementary file 2 — Supplementary Material 2. [file 12887_2025_5619_MOESM2_ESM.docx]

Supplementary File 2. Data extraction tool

| **Variable** | **Definitions and options** |
| --- | --- |
| **Study Demographics** |  |
| Study -ID | Author last name and year of publication (Umana-2023) |
| Study Title | Name or tile of study |
| CDA | Name of CDA (PECARN, BSAC and e.t.c) |
| Year of publication | Year of publication |
| Lead Authors full name |  |
| Country | List the countries involved (Spain, Ireland and e.t.c. For America use USA and united kingdom use UK). |
| Region | Europe, Aisia, Africa, Sout America and North America, Australia +, Middle East |
| Funding | Yes or No |
| **Study Characteristics** |  |
| Study Design | RCT, Case control or Cohort study |
| Method | Prospective, retrospective or combined, secondary analysis |
| CDA Analysis | Derivation, Validation or Combined |
| Method of derivation of the CDA | Recursive partitioning, expert consensus, methodology as described in the paper or cited from CDA original publication. |
| Sample size | Sample separated into derivation or validation please document as Derivation-N or Validation-N |
| Year of study period | This should reflect when patients were recruited, or retrospective data collected. |
| Number of sites involved (if multicentre) |  |
| Description of CDA low risk criteria | For example, PECARN low risk criteria is: Normal urinalysis, ANC less than 4000 cells/mm3, PCT less than 0.5 ng/ml |
| Age of infants in study | For example <90 days or 7 - 60 days and e.t.c |
| Fever without source (FWS) | Were the participants specifically FWS (Yes or No). If yes put definition. |
| Author definition of SBI |  |
| Author definition of IBI |  |
| Author definition of UTI |  |
| **Results** |  |
| Fever duration < 6hours of presentation | Number (%) |
| Gender | Male (number and %) |
| Incidence of IBI | Number (%) |
| Incidence of SBI |  |
| Incidence of meningitis | Number (%) |
| Incidence of Bacteraemia | Number (%) |
| Incidence of UTI | Number (%) |
| Diagnostic accuracy | As reported in the paper and if available |
| Sensitivity SBI | Report as % (confidence interval) |
| Specificity SBI | Report as % (confidence interval) |
| PPV SBI (positive predictive value) | Report as % (confidence interval) |
| NPV SBI (negative predictive value) | Report as % (confidence interval) |
| Sensitivity IBI | Report as % (confidence interval) |
| Specificity IBI | Report as % (confidence interval) |
| PPV IBI (Positive predictive value) | Report as % (confidence interval) |
| NPV IBI (Negative predictive value) | Report as % (confidence interval) |
| AUC Predictive modelling (Area under the curve) |  |
