# Supplementary material for: Scoping review of clinical decision aids in the assessment and management of febrile infants under 90 days of age
Source: BMC Pediatr. 2025 Apr 4;25:274. doi: 10.1186/s12887-025-05619-3 (PMC11969967; doi:10.1186/s12887-025-05619-3)
Supplement: Supplementary file 3 — Supplementary Material 3. [file 12887_2025_5619_MOESM3_ESM.docx]

Supplementary File 3. Summary of included studies

| **Study ID (number,author last name and year of pub** | **Study Title** | **CDA** | **Year of pub** | **Country** | **Region** | **Method** | **Sample size** | **Year of study period (When the study was atcually conducted)** | **Study Setting** | **Number of sites involved** | **Fever without source (Yes or no, if yes put definition)** | **Age of infants in study (e.g <90 days or 7 - 60 days)** | **Outcome (IBI, SBI or IBI+SBI= as reported in the study)** |
| --- | --- | --- | --- | --- | --- | --- | --- | --- | --- | --- | --- | --- | --- |
| **Bressan-2012** (1) | Diagnostic Performance of the Lab-score in Predicting Severe and Invasive Bacterial Infections in Well-appearing Young Febrile Infants | Lab score | 2012 | Spain and Italy | Europe | Retrospective | 1098 | 2008 - 2010 | Emergency Department | 7 | Yes | 0 - 90 days | IBI+SBI |
| **Mintegi-2014** (2) | Accuracy of a sequential approach to identify young febrile infants at low risk for invasive bacterial infection | Step-by-Step | 2014 | Spain and Italy | Europe | Retrospective | 1123 | 2009 - 2010 | Emergency Department | 7 | Yes | 0 - 90 days | IBI |
| **Gomez-2016** (3) | Validation of the "step-by-step" approach in the management of young febrile infants | Step-by-Step, Lab score, | 2016 | Spain, Italy and Switzerland | Europe | Prospective | 2185 | 2012 - 2014 | Emergency Department | 11 | Yes (FWS: Temperature measured at home or at the PED ≥38°C, in patients with a normal physical examination and no respiratory signs/symptoms or a diarrheal process.) | 0 - 90 days | IBI |
| **VIllalobos-2017** (4) | Construction of a diagnostic prediction model of severe bacterial infection in febrile infants under 3 months old | Prediction Model- Regression. | 2017 | Spain | Europe | Prospective | 702 | NR | Emergency Department | 1 | Yes | 0 - 90 days | SBI |
| **Vujevic-2017** (5) | New prediction model for diagnosis of bacterial infection in febrile infants younger than 90 days | Prediction Model- Regression | 2017 | Croatia | Europe | Retrospective | 181 | 2014 - 2015 | Not Clear | 1 | No | 0 - 90 days | SBI |
| **Aronson-2019** (6) | A Prediction Model to Identify Febrile Infants #60 Days at Low Risk of Invasive Bacterial Infection | Aronson Score (IBI Score) | 2019 | USA | North America | Retrospective | 543 | 2011 - 2016 | Emergency Department | 11 | No | 0 - 60 days | IBI |
| **Kupperman-2019** (7) | A Clinical Prediction Rule to Identify Febrile Infants 60 Days and Younger at Low Risk for Serious Bacterial Infections | PECARN | 2019 | USA | North America | Prospective | 902 (Total Sample 1821) | 2011 - 2013 | Emergency Department | 21 | No | 0 - 60 days | SBI |
| **Rabiner-2019** (8) | Validation of Risk Stratification Criteria to Identify Febrile Neonates at Low Risk of Serious Bacterial Infection | Morgan Stanley CH Protocol | 2019 | USA | North America | Retrospective | 338 | 2012 - 2014 | Emergency Department | 3 | No | 0 - 56 days | SBI |
| **Velasco-2021** (9) | Accuracy of PECARN rule for predicting serious bacterial infection in infants with fever without a source | PECARN | 2020 | SPAIN | Europe | Secondary Analysis | 1247 | 2007 - 2018 | Emergency Department | 1 | Yes | 0 - 60 days | IBI+SBI |
| **Ramgopal-2020** (10) | Machine Learning to Predict Serious Bacterial Infections in Young Febrile Infants | Prediction Model- Random forest model, Step wise regression , Support vector model, Single-hidden layer neural network. Step-by-Step, PECARN and Aronson score | 2020 | USA | North America | Secondary Analysis | 735 | 2008 - 2013 | Emergency Department | 26 | No | 0 - 60 days | SBI |
| **Nguyen-2021** (11) | Roseville Protocol for the Management of Febrile Infants 7–60 Days | Roseville Protocol, | 2021 | USA | North America | Retrospective | 2478 | 2007 - 2016 | Emergency Department | 19 | No | 7 - 60 days | IBI |
| **Chiu-2021** (12) | Using Machine Learning to Predict Invasive Bacterial Infections in Young Febrile Infants Visiting the Emergency Department | Prediction Model- Logistic regression, Support vector model, extreme gradient boosting. Aronson score | 2021 | Taiwan | Asia | Retrospective | 4211 | 2011 - 2018 | Emergency Department | 3 | No | 0 - 60 days | IBI |
| **Kuppermann-2021 (Conference paper)** (13) | Validation of a Prediction Rule for Febrile Infants Less Than or Equal to 60 Days in a Multicenter Network | PECARN | 2021 | USA | North America | Prospective | 1363 | 2016 - 2019 | Emergency Department | N/A | No | 0 - 60 days | SBI |
| **Poirier-2021 (Conference abstract)** (14) | Addition of CRP and Respiratory Viral Testing for Risk-Stratification of Febrile Young Infants | Prediction Model | 2021 | Canada | North America | Retrospective | 3461 | 2010 - 2018 | Emergency Department | 1 | No | 0 - 90 days | IBI+SBI |
| **Tsai-2021** (15) | External Validation of an Invasive Bacterial Infection Score for Young Febrile Infants | Aronson Score (IBI Score) | 2021 | USA | North America | Secondary Analysis | 4130 | 2008 - 2013 | Emergency Department | 26 | No | 0 - 60 days | IBI |
| **Waterfield-2022** (16) | Validating clinical practice guidelines for the management of febrile infants presenting to the emergency department in the UK and Ireland | NICE NG143, NICE NG51, BSAC | 2021 | UK and Ireland | Europe | Retrospective | 555 | 2018 - 2019 | Emergency Department | 6 | No | 0 - 90 days | SBI |
| **Yaeger-2021** (17) | Using Clinical History Factors to Identify Bacterial Infections in Young Febrile Infants | Prediction Model- Regression, super learner model | 2021 | USA | North America | Prospective | 877 | 2014 - 2016 | Emergency Department | 1 | No | 0 - 90 days | SBI |
| **Burstein-2022** (18) | Guideline-Based Risk Stratification for Febrile Young Infants Without Procalcitonin Measurement | AAP | 2022 | Canada | North America | Secondary Analysis | 957 | 2018 - 2021 | Emergency Department | 1 | No | 8 - 60 days | IBI |
| **Sutiman-2022** (19) | Validation and comparison of the PECARN rule, Step-by-Step approach and Lab-score for predicting serious and invasive bacterial infections in young febrile infants | PECARN, Step-by-step, Lab score | 2022 | Singapore | Asia | Prospective | 258 | 2020 - 2021 | Emergency Department | 1 | No | 0 - 90 days | IBI+SBI |
| **Yaeger-2022** (20) | Derivation of a clinical‐based model to detect invasive bacterial infections in febrile infants | Prediction Model- Regression, Super learner model | 2022 | USA | North America | Retrospective | 2311 | 2011 - 2018 | Emergency Department | 2 | No | 0 - 90 days | IBI |
| **Yaeger-2022** (21) | Refinement and validation of a clinical-based approach to evaluate young febrile infants | Prediction Model- Regression, Super learner model | 2022 | USA | North America | Secondary Analysis | 1419 | 2011 - 2018 | Emergency Department | 1 | No | 0 - 90 days | SBI |
| **Nguyen-2023** (22) | Using AAP Guidelines for Managing Febrile Infants Without C-Reactive Protein and Procalcitonin | AAP, Roseville protocol, Rochester | 2023 | USA | North America | Retrospective | 1433 | 2010 - 2019 | Emergency Department | 21 | No ( - focal bacterial infection or bronchiolitis, exclded but this didn’t include URTI, OM, ) | 8 - 60 days | IBI |
| **Umana-2023** (23) | Applying the American Academy of Pediatrics guideline to a cohort of febrile infants attending emergency departments in the UK and Ireland | AAP | 2023 | UK and Ireland | Europe | Secondary Analysis | 536 | 2018 - 2019 | Emergency Department | 6 | No | 8 - 90 days | IBI |
| **Yaeger-2023** (24) | Performance of AAP Clinical Practice Guideline for Febrile Infants at One Pediatric Hospital | AAP | 2023 | USA | North America | Secondary Analysis | 507 | 2011 - 2018 | Emergency Department | 1 | No | 8 - 90 days | IBI |
| **Chong-2023** (25) | Febrile infants risk score at triage (FIRST) for the early identification of serious bacterial infections | FIRST, FIRST+ | 2023 | Singapore | Asia | Prospective | 1002 | 2018 - 2021 | Emergency Department | 1 | No | 0 - 90 days | SBI |
| **Yang-2023** (26) | Explainable deep learning model to predict invasive bacterial infection in febrile young infants: A retrospective study | Prediction Model- Deep Learning Model. Aronson score, Step-by-Step | 2023 | Taiwan | Asia | Retrospective | 1847 | 2011 - 2019 | Emergency Department | 1 | No | 0 - 60 days | IBI |
| **Greenhow-2023** (27) | CA FIRST (California Febrile Infant Risk Stratification Tool) Algorithm Development in a Learning Health System | CA FIRST | 2023 | USA | North America | Retrospective | 1412 | 2022 - 2022 | Emergency Department | 21 | No | 7 - 60 days (modified 7 - 90 days) | IBI |
| **Chong-2023** (28) | Adding heart rate n-variability (HRnV) to clinical assessment potentially improves prediction of serious bacterial infections in young febrile infants at the emergency department: a prospective observational study | Prediction Model - Regression | 2023 | Singapore | Asia | Prospective | 312 | 2017 - 2021 | Emergency Department | 1 | No | 0 - 90 days | SBI |
| **Ballard-2024**  (29) | An all-inclusive model for predicting invasive bacterial infection in febrile infants age 7–60 days | Prediction Model – Regression. Machine learning tree-based | 2024 | USA | North America | Retrospective | 4411 | 2010 - 2019 | Emergency Department | 37 | No | 7 – 60 days | IBI |
| **Knudson-2024**  (30) | Performance of AAP CPG for Ineligible WellAppearing Febrile Infants Aged 8–60 Days | AAP | 2024 | USA | North America | Secondary Analysis | 648 | 2011 - 2018 | Emergency Departments | 1 | No | 8 – 60 days | IBI |
| **Umana-2024**  (31) | Performance of clinical decision aids (CDA) for the care of young febrile infants: a multicentre prospective cohort study conducted in the UK and Ireland | BSAC  AAP  NICE NG143  Aronson Score | 2024 | UK and Ireland | Europe | Prospective | 1821 | 2022 - 2023 | Emergency Department | 35 | No | < 90 days | IBI |
| **Burstein-2025**  (32) | Optimizing Management of Febrile  Young Infants Without Serum  Procalcitonin | AAP  AAP derived thresholds | 2025 | Canada | North America | Secondary Analysis | 1987 | 2018 - 2023 | Emergency Department | 1 | No | 8 – 60 days | IBI |

**References**

1. Bressan S, Gomez B, Mintegi S, Da Dalt L, Blazquez D, Olaciregui I, et al. Diagnostic performance of the Lab-score in predicting severe and invasive bacterial infections in well-appearing young febrile infants. Pediatric Infectious Disease Journal. 2012;31(12):1239–44.

2. Mintegi S, Bressan S, Gomez B, Da Dalt L, Blázquez D, Olaciregui I, et al. Accuracy of a sequential approach to identify young febrile infants at low risk for invasive bacterial infection. Emergency Medicine Journal. 2013;31(e1):e19–24.

3. Gomez B, Mintegi S, Bressan S, Da Dalt L, Gervaix A, Lacroix L. Validation of the ‘step-by-step’ approach in the management of young febrile infants. Pediatrics. 2016;138(2).

4. Villalobos Pinto E., Sanchez-Bayle M. Construction of a diagnostic prediction model of severe bacterial infection in febrile infants under 3 months old. An Pediatr. 2017;87(6):330–6.

5. Vujevic M, Benzon B, Markic J. New prediction model for diagnosis of bacterial infection in febrile infants younger than 90 days. TurkJPediatr. 2017;59(3):261.

6. Aronson PL, Shabanova V, Shapiro ED, Wang ME, Nigrovic LE, Pruitt CM, et al. A prediction model to identify febrile infants≤ 60 days at low risk of invasive bacterial infection. Pediatrics. 2019;144(1).

7. Kuppermann N, Dayan PS, Levine DA, Vitale M, Tzimenatos L, Tunik MG, et al. A Clinical Prediction Rule to Identify Febrile Infants 60 Days and Younger at Low Risk for Serious Bacterial Infections. JAMA Pediatrics. 2019;173(4):342–51.

8. Rabiner JE, Capua M, Golfeiz D, Shoag J, Avner JR. Validation of Risk Stratification Criteria to Identify Febrile Neonates at Low Risk of Serious Bacterial Infection. Glob pediatr health. 2019;6(101670224):2333794X19845076.

9. Velasco R, Gomez B, Benito J, Mintegi S. Accuracy of PECARN rule for predicting serious bacterial infection in infants with fever without a source. Archives of Disease in Childhood. 2021;106(2):143–8.

10. Ramgopal S, Horvat CM, Yanamala N, Alpern ER. Machine learning to predict serious bacterial infections in young febrile infants. Pediatrics. 2020;146(3).

11. Nguyen THP, Young BR, Poggel LE, Alabaster A, Greenhow TL. Roseville Protocol for the Management of Febrile Infants 7–60 Days. Hospital Pediatrics. 2021 Jan 1;11(1):52–60.

12. Chiu I, Cheng C, Zeng W, Huang Y, Lin C. Using Machine Learning to Predict Invasive Bacterial Infections in Young Febrile Infants Visiting the Emergency Department. JOURNAL OF CLINICAL MEDICINE. 2021 May;10(9).

13. Kuppermann N., Dayan P.S., Atabaki S., Bogie A., Cator A., Cohen D., et al. Validation of a prediction rule for serious bacterial infections(SBIS) in febrile infants < 60 days in a multicenter network. Pediatrics. 2021;147(3):513–5.

14. Poirier C, Filion-Ouellet E, Anderson G, Burstein B. 107 Addition of CRP and Respiratory Viral Testing for Risk-Stratification of Febrile Young Infants. Paediatrics & Child Health. 2021 Oct 1;26(Supplement_1):e75–6.

15. Tsai S.J., Ramgopal S. External validation of an invasive bacterial infection score for young febrile infants. Hosp Pediatr. 2021;11(3):239–44.

16. Waterfield T, Lyttle MD, Munday C, Foster S, McNulty M, Platt R, et al. Validating clinical practice guidelines for the management of febrile infants presenting to the emergency department in the UK and Ireland. Arch Dis Child. 2022 Apr;107(4):329–34.

17. Yaeger JP, Jones J, Ertefaie A, Caserta MT, van Wijngaarden E, Fiscella K. Using Clinical History Factors to Identify Bacterial Infections in Young Febrile Infants. In: J Pediatr [Internet]. Mosby Inc.; 2021. p. 192-199.e2. Available from: https://www.scopus.com/inward/record.uri?eid=2-s2.0-85100626346&doi=10.1016%2fj.jpeds.2020.12.079&partnerID=40&md5=2fa8bccc01dfea4db474a9295c6004a7

18. Burstein B, Alathari N, Papenburg J. Guideline-Based Risk Stratification for Febrile Young Infants Without Procalcitonin Measurement. Pediatrics. 2022;149(6):6–8.

19. Sutiman N, Khoo ZX, Ong GYK, Piragasam R, Chong SL. Validation and comparison of the PECARN rule, Step-by-Step approach and Lab-score for predicting serious and invasive bacterial infections in young febrile infants. Ann Acad Med Singap. 2022 Oct 26;51(10):595–604.

20. Yaeger JP, Jones J, Ertefaie A, Caserta MT, Fiscella KA. Derivation of a clinical-based model to detect invasive bacterial infections in febrile infants. Journal of Hospital Medicine. 2022;17(11):893–900.

21. Yaeger JP, Jones J, Ertefaie A, Caserta MT, van Wijngaarden E, Fiscella K. Refinement and validation of a clinical-based approach to evaluate young febrile infants. Hospital Pediatrics. 2022;12(4):399–407.

22. Nguyen THP, Young BR, Alabaster A, Vinson DR, Mark DG, Van Winkle P, et al. Using AAP Guidelines for Managing Febrile Infants Without C-Reactive Protein and Procalcitonin. Pediatrics. 2023 Jan 1;151(1):e2022058495.

23. Umana E, Norman-Bruce H, Mills C, Mitchell H, McFetridge L, Waterfield T, et al. Applying the American Academy of Pediatrics guideline to a cohort of febrile infants attending emergency departments in the UK and Ireland. European Journal of Emergency Medicine. 2023 Jun;30(3):219–21.

24. Yaeger JP, Richfield C, Schiller E, Oh Y, Pereira BMC, Shabangu T, et al. Performance of AAP Clinical Practice Guideline for Febrile Infants at One Pediatric Hospital. Hospital Pediatrics. 2023 Feb 2;13(3):e47–50.

25. Chong SL, Niu C, Ong GYK, Piragasam R, Khoo ZX, Koh ZX, et al. Febrile infants risk score at triage (FIRST) for the early identification of serious bacterial infections. Sci Rep. 2023 Sep 22;13(1):15845.

26. Yang Y, Wang YM, Lin CHR, Cheng CY, Tsai CM, Huang YH, et al. Explainable deep learning model to predict invasive bacterial infection in febrile young infants: A retrospective study. International Journal of Medical Informatics. 2023 Apr;172:105007.

27. Greenhow T.L., Nguyen T.H., Young B.R., Somers M.J., Huang J., Alabaster A., et al. CA FIRST (California Febrile Infant Risk Stratification Tool) Algorithm Development in a Learning Health System. Perm J. 2023;((Greenhow) Division of Infectious Diseases, Department of Pediatrics, Kaiser Permanente Northern California, San Francisco, CA, United States):1–7.

28. Chong SL, Niu C, Piragasam R, Koh ZX, Guo D, Lee JH, et al. Adding heart rate n-variability (HRnV) to clinical assessment potentially improves prediction of serious bacterial infections in young febrile infants at the emergency department: a prospective observational study. Annals of Translational Medicine. 2023 Jan 15;11(1):6–6.

29. Ballard DW, Huang J, Sharp AL, Mark DG, Nguyen THP, Young BR, et al. An all-inclusive model for predicting invasive bacterial infection in febrile infants age 7–60 days. Pediatr Res. 2024 Aug;96(3):759–65.

30. Knudson MJ, Yaeger JP. Performance of AAP CPG for Ineligible Well-Appearing Febrile Infants Aged 8–60 Days. Hospital Pediatrics. 2024 Dec 1;14(12):e509–12.

31. Umana E, Mills C, Norman–Bruce H, Mitchell H, McFetridge L, Lynn F, et al. Performance of clinical decision aids (CDA) for the care of young febrile infants: a multicentre prospective cohort study conducted in the UK and Ireland. eClinicalMedicine [Internet]. 2024 Dec 1 [cited 2024 Dec 12];78. Available from: https://www.thelancet.com/journals/eclinm/article/PIIS2589-5370(24)00540-6/fulltext

32. Burstein B, Wolek C, Poirier C, Yannopoulos A, Charles Casper T, Kaouache M, et al. Optimizing Management of Febrile Young Infants Without Serum Procalcitonin. Pediatrics. 2025 Jan 2;e2024068200.
