# Supplementary material for: Scoping review of clinical decision aids in the assessment and management of febrile infants under 90 days of age
Source: BMC Pediatr. 2025 Apr 4;25:274. doi: 10.1186/s12887-025-05619-3 (PMC11969967; doi:10.1186/s12887-025-05619-3)
Supplement: Supplementary file 4 — Supplementary Material 4. [file 12887_2025_5619_MOESM4_ESM.docx]

Supplementary File 4. Studies published per year
