# Supplementary material for: Scoping review of clinical decision aids in the assessment and management of febrile infants under 90 days of age
Source: BMC Pediatr. 2025 Apr 4;25:274. doi: 10.1186/s12887-025-05619-3 (PMC11969967; doi:10.1186/s12887-025-05619-3)
Supplement: Supplementary file 5 — Supplementary Material 5. [file 12887_2025_5619_MOESM5_ESM.docx]

Supplementary File 2. CDAs and variable components

| **Model or risk criteria CDA** | **CDA** | **Age** | **Medical History** | **Gender** | **Duration of fever/illness** | **Appearance** | **Gestational age** | **Deprivation score/insurance** | **Temperature/Tmax** | **Heart Rate (HR)** | **Respiratory Rate (RR)** | **HR Variability** | **Urinalysis** | **PCT** | **CRP** | **Neutrophil Count** | **Total White Blood Cells** | **Band Cells (full count or ratio)** | **Basophils** | **Eosinoiphils** | **Lymphocytes** | **Monocytes** | **Platelets** | **Other bloodtes (glucose / liver fucntion)** | **Respiratory Virus Tests** | **Cough /signs of bronchiolitis** |
| --- | --- | --- | --- | --- | --- | --- | --- | --- | --- | --- | --- | --- | --- | --- | --- | --- | --- | --- | --- | --- | --- | --- | --- | --- | --- | --- |
| CDA | NICE NG51 | 1 |  |  |  |  |  |  |  |  |  |  |  |  |  |  |  |  |  |  |  |  |  |  |  |  |
| CDA | Lab Score |  |  |  |  |  |  |  |  |  |  |  | 1 | 1 | 1 |  |  |  |  |  |  |  |  |  |  |  |
| CDA | NICE NG143 | 1 |  |  |  | 1 |  |  |  |  |  |  |  |  |  |  | 1 |  |  |  |  |  |  |  |  |  |
| CDA | PECARN |  |  |  |  |  |  |  |  |  |  |  | 1 | 1 |  | 1 |  |  |  |  |  |  |  |  |  |  |
| CDA | AAP V2 |  |  |  |  |  |  |  | 1 |  |  |  |  |  | 1 | 1 |  |  |  |  |  |  |  |  |  |  |
| CDA | Aronson Score (IBI Score) | 1 |  |  |  |  |  |  | 1 |  |  |  | 1 |  |  | 1 |  |  |  |  |  |  |  |  |  |  |
| CDA | BSAC | 1 |  |  |  | 1 |  |  |  |  |  |  | 1 |  | 1 |  |  |  |  |  |  |  |  |  |  |  |
| CDA | CA FIRST (California Febrile Infant Risk Stratification Tool) Algorithm | 1 |  |  |  | 1 |  |  |  |  |  |  | 1 |  |  |  |  |  |  |  |  |  |  |  |  | 1 |
| CDA | Morgan Stanley CH Protocol |  |  |  |  |  |  |  |  |  |  |  | 1 |  |  |  | 1 | 1 |  |  |  |  |  | 1 |  |  |
| Model | Ramgopal 2020 - Step Wise Regression |  |  |  |  |  |  |  |  |  |  |  | 1 | 1 |  | 1 | 1 |  |  |  |  |  |  |  |  |  |
| Model | Ramgopal 2020 - Random Forest Modelling |  |  |  |  |  |  |  |  |  |  |  | 1 | 1 |  | 1 | 1 |  |  |  |  |  |  |  |  |  |
| Model | Ramgopal 2020 - Support Vector Maching Model |  |  |  |  |  |  |  |  |  |  |  | 1 | 1 |  | 1 | 1 |  |  |  |  |  |  |  |  |  |
| Model | Ramgopal 2020 - Single-Hidden Layer Neural Network |  |  |  |  |  |  |  |  |  |  |  | 1 | 1 |  | 1 | 1 |  |  |  |  |  |  |  |  |  |
| Model | Vujevic 2017 - Regression Model |  |  | 1 |  |  |  |  | 1 |  |  |  |  |  | 1 |  | 1 |  |  |  |  |  |  |  |  |  |
| Model | Chong 2022 - FIRST Model | 1 |  | 1 | 1 |  |  |  | 1 |  |  |  |  |  |  |  |  |  |  |  |  |  |  |  |  |  |
| Model | Poirier 2021 - Model |  |  |  |  |  |  |  |  |  |  |  | 1 |  | 1 |  | 1 |  |  |  |  |  |  |  | 1 |  |
| CDA | AAP | 1 |  |  |  | 1 |  |  | 1 |  |  |  |  |  | 1 | 1 |  |  |  |  |  |  |  |  |  |  |
| CDA | Roseville | 1 |  |  |  |  |  |  | 1 |  |  |  | 1 |  |  |  | 1 | 1 |  |  |  |  |  |  |  |  |
| Model | Chiu 2021 - Support Vector Maching Model |  |  |  |  |  |  |  |  | 1 |  |  |  |  | 1 | 1 |  | 1 | 1 |  |  |  |  |  |  |  |
| Model | Yang 2023 - Deep Learning Model | 1 |  |  |  |  |  |  | 1 | 1 |  |  |  |  | 1 | 1 |  |  |  |  |  |  |  |  |  |  |
| Model | Chong 2022 - Regression Model | 1 |  | 1 | 1 |  |  |  |  |  | 1 | 1 |  |  |  |  |  |  |  |  |  |  |  |  |  |  |
| Model | Ballard 24, LASSO | 1 | 1 |  |  |  | 1 |  | 1 |  |  |  | 1 |  |  |  |  |  |  |  |  |  |  |  |  |  |
| Model | Ballard 24, Logistic | 1 | 1 |  |  |  | 1 |  | 1 |  |  |  | 1 |  |  |  |  |  |  |  |  |  |  |  |  |  |
| Model | Ballard 24, Random Forest | 1 | 1 |  |  |  | 1 |  | 1 |  |  |  | 1 |  |  |  |  |  |  |  |  |  |  |  |  |  |
| Model | Ballard 24, XGBoost | 1 | 1 |  |  |  | 1 |  | 1 |  |  |  | 1 |  |  |  |  |  |  |  |  |  |  |  |  |  |
| CDA | step by step | 1 |  |  |  | 1 |  |  |  |  |  |  | 1 | 1 | 1 | 1 |  |  |  |  |  |  |  |  |  |  |
| Model | Chong 2022 - FIRST+ Model | 1 |  | 1 | 1 |  |  |  | 1 |  |  |  | 1 | 1 |  |  |  |  |  |  |  |  |  |  |  |  |
| Model | Chiu 2021 - Extreme Gradient Boosting |  |  |  |  |  |  |  |  | 1 |  |  |  |  | 1 | 1 | 1 | 1 |  | 1 |  | 1 |  |  |  |  |
| Model | Villalobos 2017 - Regression Model |  | 1 |  |  | 1 |  |  | 1 |  |  |  | 1 | 1 | 1 | 1 | 1 |  |  |  |  |  |  |  |  |  |
| Model | Chiu 2021 - Logistic Regression Model | 1 |  |  |  |  |  |  | 1 |  |  |  |  |  | 1 | 1 |  | 1 | 1 |  | 1 |  | 1 |  |  |  |
| Model | Yaeger 2021 & 2022 - Regression Model | 1 | 1 | 1 | 1 | 1 | 1 | 1 | 1 |  |  |  | 1 |  |  |  |  |  |  |  |  |  |  |  |  | 1 |
| Model | Yaeger 2021 & 2022 - Super Learner model | 1 | 1 | 1 | 1 | 1 | 1 | 1 | 1 |  |  |  | 1 |  |  |  |  |  |  |  |  |  |  |  |  | 1 |
